# Exercise stress and tissue remodeling: advances in exosome-mediated RNA–RBP networks in musculoskeletal injury repair and functional recovery

**DOI:** 10.3389/fgene.2026.1775005

**Published:** 2026-03-11

**Authors:** Junjie Liu, Heming Chen, Yupeng Yang, Zheheng Jia, Ying Li, Xintong Zhong, Huimei Jiang, Zhujun Mao, Mi Zheng

**Affiliations:** 1 Graduate School, Harbin Sport University, Harbin, Heilongjiang, China; 2 College of Science and Technology, China Three Gorges University, Yichang, China; 3 School of Basic Medical Sciences, China Three Gorges University, Yichang, China

**Keywords:** biomarkers, circular RNA, engineered exosomes, exercise stress, exosomes, functional recovery, musculoskeletal injury, RNA-binding proteins (RBPs)

## Abstract

Exercise is a double-edged sword. It can either cause adaptive remodeling of musculoskeletal tissues or lead to acute or chronic injury. Exosomes that are boosted with circular RNAs (circRNAs), long non-coding RNAs (lncRNAs), and RNA-binding proteins (RBPs) have been a focus in recent years as important contributors to the process of repair after exposure to stress during exercise. This narrative review is a summarization of the impacts of different types and intensities of exercise modalities on musculoskeletal patterns of RNA expression and exosome secretion. It also describes the role of exosomes that initiate RNA–RBP networks that coordinate regenerative and inflammatory reactions in recipient cells. The article notes the promise of these networks as diagnostic biomarkers and therapeutic targets and also suggests new clinical uses, such as engineered exosome-based interventions. This review will build a holistic but theoretically sound framework to inform future direct-to-patient research in exercise medicine by taking into account multi-omics data and its functional validation. The purpose of this article is to present better diagnostic and rehabilitative approaches to musculoskeletal injuries, leading to suitable functional recovery outcomes.

## 1 Introduction

The musculoskeletal system facilitates movement, supports the body’s structure, and maintains personal physical functionality. Exercise acts as a vital physiological trigger, inducing adaptive remodeling of musculoskeletal tissues, including muscle, bone, and tendon. Exercise produces several desirable effects, including strengthening muscles, promoting bone density, and developing optimal joint function. These effects contribute to maintaining health and preventing diseases throughout life (Kargl et al., 2025; Miko et al., 2020; O'Leary et al., 2021; Terryn et al., 2021). However, the relationship between exercise and musculoskeletal health is not straightforward. Moderate exercise can lead to adaptive remodeling, while excessive or unaccustomed mechanical loading may cause various musculoskeletal injuries, ranging from acute trauma to chronic overuse syndrome. These injuries not only impair physical activity but also significantly reduce quality of life and may have socioeconomic implications (Cranswick et al., 2025; Harper-Hanigan and Gruber, 2022; Traweger et al., 2025). Therefore, understanding the mechanisms underlying exercise-induced tissue remodeling and injury repair is essential to optimize rehabilitation and functional recovery (Chen et al., 2024; Cherief et al., 2023; Radovanović et al., 2022).

Traditionally, musculoskeletal biology studies have focused on intracellular signaling networks of cellular responses to mechanical stimuli. Mechanisms such as the PI3K–AKT signaling cascade’s role in muscle hypertrophy and osteogenic differentiation have been elucidated (Liu et al., 2025; Wang Q et al., 2021), as has the role of inflammatory cytokines and growth factors in tendon repairs (Alsereidi et al., 2024; Chen et al., 2025; Otzel et al., 2021). Nonetheless, the effective processes involved in repair and remodeling, and the coordination between tissue-level responses, cannot be fully attributed to these intracellular mechanisms alone. It is increasingly apparent that intercellular communication plays a significant role in the coordination of the sophisticated cellular interactions to accomplish tissue regeneration (Avalos and Forsthoefel, 2022; Li et al., 2023; Magaye et al., 2021). Intercellular interactions of different cell types within the musculoskeletal niche, including morphine cells, immune cells, fibroblasts, endothelial cells, and osteoblasts, mediate the inflammatory response, extracellular matrix remodeling, and angiogenesis, all of which play a key role in successful tissue repair (Döding et al., 2025; Jung et al., 2024; Lynch et al., 2024; Sadiq et al., 2025). Despite this understanding, the processes that underlie intercellular signaling in states of exercise-induced stress and musculoskeletal injury have not been fully comprehended.

Extracellular vesicles, especially exosomes, are important intercellular communication mediators in a variety of physiological and pathological processes. Exosomes, which are lipid bilayer vesicles approximately at the nanoscale, are produced by virtually all types of cells. They contain a functional cargo of biomolecules, including proteins, lipids, messenger RNAs, microRNAs, and other non-coding RNAs (Miron et al., 2024; Ruan et al., 2021). These vesicles can transmit molecular signals between cells, thereby shaping the behavior of recipient cells and integrating tissue responses. Recent studies have shown the importance of exosome-based RNA and RNA-binding protein (RBP) networks in the regulation of cell proliferation, differentiation, extracellular matrix remodeling, and s inflammation in the process of tissue repair (Liu and Wu, 2025; Zhu et al., 2025). Exosomes formed by stem cells or resident cells in musculoskeletal tissues have been reported to drive the regeneration process by boosting the efficiency of satellite cells, regulating immune homeostasis, and triggering angiogenesis (Huang et al., 2023; Miron et al., 2024). In addition, the exosomal cargo composition actively responds to exercise-provoked stress and injury, and it is proposed to mediate adaptive remodeling and functional recovery (Sadiq et al., 2025).

With these developments, there is now a significant gap in the incorporation of the knowledge about musculoskeletal adaptation in exercise and the newly available insights into the exosome-mediated intercellular signaling networks. Conventional paradigms of injury repair have largely ignored the roles played by extracellular vesicles and their RNA–RBP content in organizing cellular events in response to mechanical and metabolic stress conditions. Such a gap narrows the possibilities of developing specific therapeutic measures by utilizing or controlling exosome pathways to promote tissue regeneration and functional rehabilitation. To overcome this knowledge gap, a systematic study of the exosome-mediated RNA–RBP interactions in response to exercise stress and how they mediate mechanochemical processes in musculoskeletal injury repair and recovery needs to be undertaken.

For this reason, we examine the latest developments in exosome-mediated RNA–RBP networks for repairing both musculoskeletal tissue and promoting functional rehabilitation following exercise-induced stress. We combine current research on the effects of exercise on exosomal cargo, the molecular actions of exosomes on RNA and RBPs, and their subsequent effects on muscle, bone, and tendon repair. We also examine the clinical translation potential of exosome RNA–RBP network targeting to maximize rehabilitation and restore musculoskeletal activity. We believe that this review can bridge the gap between basic molecular processes and clinical interventions, thereby advancing exercise physiology and regenerative medicine by integrating fundamental biological insights with clinical practice.

## 2 Exercise-induced exosomes: mediating systemic adaptation and tissue repair through intercellular RNA communication

### 2.1 Effects of different exercise modes and intensities on cellular RNA expression profiles

Muscle satellite cells, fibroblasts, and immune cells are among the key cell types that are metabolically and mechanically affected by exercise-induced molecular responses in musculoskeletal tissues. Eccentric and concentric exercises (i.e., lengthening and shortening muscle contractions), along with endurance and explosive power training modalities, exert unique regulatory effects on the transcriptomes of these cells (Brown et al., 2022; Khuu et al., 2021). As an illustration, eccentric contractions often cause microdamage to muscle fibers, activating satellite cells that upregulate repair- and regeneration-related genes, including immediate early genes such as cFos, which has been shown to increase following photobiomodulation in exercised muscles, suggesting the involvement of both satellite and interstitial cells in muscle repair (Cheema et al., 2025; Garella et al., 2025; Squecco et al., 2021). Conversely, concentric exercise is mainly defined by metabolic adjustments, less apparent muscle rupture, the remodeling of gene systems involved in mitochondrial biogenesis, and oxidative metabolism. Endurance training provokes certain changes in long non-coding RNAs (lncRNAs), as evidenced by transcriptomic studies that show the presence of specific lncRNA expression patterns following 8 weeks of endurance training. The process involves such lncRNAs related to autophagy, angiogenesis, and extracellular matrix remodeling (Bonilauri and Dallagiovanna, 2020; Touron et al., 2020; Touron et al., 2022). Both high-intensity interval training (HIIT) and resistance training induce unique lncRNA signatures, with the HIIT generating the most comprehensive differential expressions, suggesting modality-specific patterns of non-coding RNA networks. In addition, immune cells that penetrate skeletal muscle during exercise have altered RNA profiles that indicate their functions in resolving inflammation and remodeling (Carrasco-Poyatos et al., 2024; Riedel et al., 2025). CircRNAs and lncRNAs have additional effects on gene expression; these effectors of functional diversity serve as microRNA sponges, transcriptional regulators, or scaffolds for RNA-binding proteins (RBPs), depending on the intensity and duration of exercise. An example of this is circulating piRNAs, which have been found to respond to acute exercise sessions and are related to fitness parameters, including maximal oxygen uptake (V̇O_2_max), and therefore should be considered biomarkers of exercise adaptation (Bilberg et al., 2024; Hov et al., 2023; Riedel et al., 2025; van Baak et al., 2021).

**FIGURE 1:**
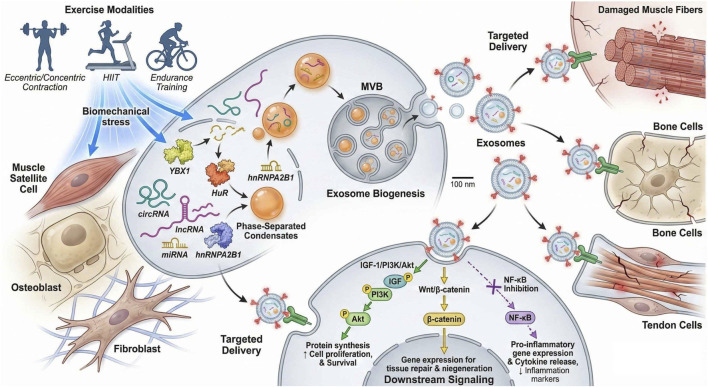
Exercise-induced exosomal RNA–RBP network mediates musculoskeletal tissue repair and regeneration. The schematic depicts exosome biogenesis under exercise stress, selective RNA–RBP cargo sorting, targeted delivery to recipient cells, and downstream activation of repair-related signaling pathways.

Collectively, these findings indicate that the type and intensity of exercise intricately regulate RNA expression in musculoskeletal cells. This regulation governs complex molecular pathways, ultimately facilitating tissue adaptation, repair, and functional recovery. Table 1 provides a comprehensive outline of the regulatory outcomes of different exercise types/intensities on RNA expression and exosome secretion (Bizjak et al., 2021; Gaffney et al., 2021; Willis et al., 2021). Exercise-induced biomechanical stress triggers a mechanistic cascade of events, including exosome biogenesis, RNA–RBP sorting, and targeted delivery to damaged musculoskeletal cells, visualized in Figure 1, as an integrated whole-body representation of the exercise–exosome–RNA axis mechanism of molecular regulation.

**TABLE 1:** Regulation of RNA expression and exosome secretion in musculoskeletal tissues by different exercise modes/intensities.

Exercise type/Intensity	Core-regulated RNA type	Target cell/Tissue	Biological effects	Molecular mechanisms	Reference
Eccentric exercise (muscle lengthening contractions)	cFos (immediate early gene), circRNAs	Muscle satellite cells and muscle fibers	Induces muscle fiber microdamage repair and activates satellite cell proliferation and differentiation	Triggers damage-related signaling pathways and upregulates transcription of repair-associated genes	Cheema et al. (2025)
Concentric exercise (muscle shortening contractions)	Mitochondrial biogenesis-related mRNAs and lncRNAs	Skeletal muscle cells	Promotes metabolic adaptation and increases oxidative metabolic capacity	Regulates the expression of gene networks associated with mitochondrial function	Bonilauri and Dallagiovanna (2020)
Endurance training (8-week continuous intervention)	Specific lncRNAs (autophagy/angiogenesis-related)	Skeletal muscle and vascular endothelial cells	Improves autophagic activity, promotes angiogenesis, and regulates extracellular matrix remodeling	lncRNAs act as miRNA sponges or transcriptional regulators	Bonilauri and Dallagiovanna (2020)
High-intensity interval training (HIIT)	Differentially expressed lncRNAs (broadest coverage) and piRNAs	Skeletal muscle and immune cells	Induces significant transcriptomic reprogramming and reflects exercise adaptation levels	Regulates signaling pathways related to inflammation resolution and tissue repair	Riedel et al. (2025)
Resistance training	Myogenesis-related lncRNAs and circRNAs	Muscle satellite cells and myoblasts	Promotes muscle hypertrophy and increases muscle strength	Activates the IGF-1/PI3K/Akt pathway and regulates myogenic differentiation genes	Chen et al. (2020)
Acute exercise (single exercise bout)	Circulating piRNAs	Multiple systemic tissues (skeletal muscle and cardiovascular system)	Correlates with maximal oxygen uptake (VO_2max_) and serves as a biomarker of exercise adaptation	Participates in cellular stress response and metabolic regulatory networks	Riedel et al. (2025)

Summarizes the key regulatory effects of exercise modalities/intensities on RNA expression and exosome secretion. The data are derived from functional experiments and peer-reviewed literature, covering core RNA types and their roles in tissue adaptation and repair.

### 2.2 Biogenesis of exosomes and selective RNA packaging mechanisms under exercise stress

Exosomes are nanoscale extracellular vesicles produced as a result of the endosomal pathway, that is, the inward budding of multivesicular bodies (MVBs), which then fuse with the plasma membrane to excrete intraluminal vesicles as exosomes. Stress in cells that occurs during exercise influences the biogenesis and release of exosomes, thereby increasing exosome secretion into an intercellular signaling network (Mérida-Cerro et al., 2025; Patel et al., 2022; Weng et al., 2023). The biogenesis of this process entails the use of ESCRT-dependent and -independent pathways to transport the endosomal sorting complex and regulate cargo selection and vesicle formation (Marie et al., 2023; Maurya et al., 2022). Recent transcriptomic and mechanistic findings have indicated that classes of RNA, such as circRNAs and lncRNAs, along with RBPs, are specifically packaged into exosomes and create particular molecular signatures that characterize the physiological status of the releasing cells (Liu J et al., 2021; Yang et al., 2025; Yen et al., 2025). As an example, the liquid phase separation of the RNA-binding protein YBX1 generates condensates that selectively attenuate microRNAs such as miR-223 to be incorporated into exosomes, illustrating the complex process of RNA cargo selection done using a phase-separated RBP–RNA complex (Green et al., 2023; Liu X. M. et al., 2021). Moreover, autophagosome fusion with MVBs under the conditions of viral infection specifies the sorting of the viral RNAs into the exosomes, which sheds light on the role of the interdependence between the autophagy and exosome pathways in the sorting of RNAs (Bertolio et al., 2025; Zhang et al., 2022).

**FIGURE 2:**
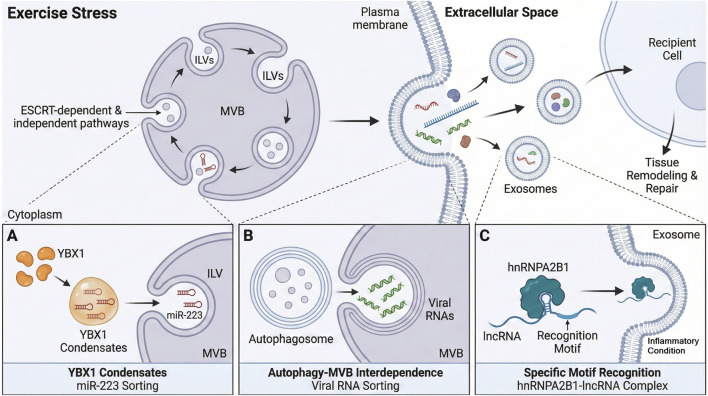
Biogenesis of exosomes and selective RNA packaging mechanisms under exercise stress. The schematic illustrates exercise-enhanced exosome biogenesis via ESCRT-dependent and independent MVB pathways under stress, alongside specific selective RNA cargo packaging mechanisms, shown in the lower panels: **(A)** YBX1 liquid phase separation sorting miR-223, **(B)** Autophagy-MVB fusion sorting viral RNAs, and **(C)** hnRNPA2B1 motif recognition loading lncRNAs for tissue repair.

Exosomal RNA cargo is not selected by chance but is discontinuously concentrated in regulatory non-coding RNAs that regulate the functionality of the recipient cell. The selective packaging entails recognition motifs and communication with RBPs, including hnRNPA2B1, involved in loading particular lncRNAs into exosomes observed in airway epithelial cells under inflammatory conditions (Hu et al., 2024; Liu et al., 2022; Liu Z et al., 2024; Song et al., 2024; Wu et al., 2025). The above molecular processes guarantee that exosomes transfer specific RNA–RBP complexes that are precise signaling units, permitting specific communication to take place during exercise-mediated tissue remodeling and repair. Figure 2 is a visual representation of these molecular processes, illustrating exercise-induced biogenesis of exosomes via MVB pathways and distinct mechanisms of selective RNA–RBP cargo sorting.

### 2.3 Targeted delivery of exercise-induced exosomes among tissues

Exercise-induced exosomes are delivered to local and distant tissues through targeted mechanisms, enabling intercellular communication critical for musculoskeletal repair; this enables intercellular communication that is important for musculoskeletal repair and functional recovery. The specificity of the targeting is facilitated by the surface molecules on the exosomes that bind to receptors present on receiver cells and facilitate specific uptake in damaged muscle fibers, inflammatory cells, or osteogenic cells (Ionescu et al., 2025; Lee et al., 2024; Li et al., 2021; Sič et al., 2025). An example is the case of exosomes released by exercised muscles or the adjacent stromal cells that transport RNA and protein cargo that alters the behavior of the recipient cells, facilitating regeneration and resolving inflammation (Du et al., 2025; Huang et al., 2024). The efficacy of exosomal RNAs to mediate exercise-induced physiological effects in animal models and organoid systems has been experimentally validated, and exosomal RNAs have been shown to effectively modify gene expression and cellular phenotypes in target tissues (Barthelaix et al., 2025; Pajarskienė et al., 2025). The RNA profiles of plasma exosomes that are purified from exercised rodents show some changes, such as the presence of upregulated neuroprotective mRNA and miRNA that correlate with enhanced tissue function and reduced injury (Citron et al., 2025; Fei et al., 2024).

Exosomes are taken up by recipient cells by endocytosis or membrane fusion, although immune cells and osteoblasts respond to exosomal signals by signaling through regenerative pathways. Moreover, exosomes can regulate cell populations involved in inflammation. For example, exercise has been shown to decrease pro-inflammatory macrophages in liver models, in part via exosome-mediated signaling (Akhtar et al., 2025; Fredrickson et al., 2021; von Kaeppler et al., 2021). These results indicate that exercise-induced exosomes play a central role in the delivery of RNA–RBP networks that coordinate tissue-specific responses to organize repair and adaptation processes across musculoskeletal compartments.

## 3 Molecular mechanisms of exosome-mediated RNA–RBP networks in musculoskeletal injury repair

### 3.1 Roles of circRNAs and lncRNAs as molecular sponges and scaffolds

CircRNAs and lncRNAs are proposed to play central roles in musculoskeletal injury repair by acting as molecular sponges of microRNAs (miRNAs) and scaffolds for RBP complexes, respectively. These roles are largely based on correlative studies and *in vitro* evidence (Li X et al., 2024; Liang et al., 2025). Direct *in vivo* experimental validation is still needed to confirm their specific contributions to tissue repair. Both classes of circRNAs and lncRNAs share the property of miRNA sequestration via complementary binding, which has the effect of reducing miRNA binding to its target mRNAs, deregulating essential regenerative genes (Ni et al., 2023; Wang J et al., 2021; Zhang Y et al., 2023). As an example, the miRNA sponging mediated by circRNAs/lncRNAs controls the insulin-like growth factor 1 (IGF-1)/PI3K/Akt pathway, which is a central axis that fosters muscle and bone regeneration. CircRNAs and lncRNAs promote IGF-1 signaling by absorbing the miRNAs that target components of this pathway, thereby promoting the cell growth and survival needed for tissue repair (Chen et al., 2020; Kong et al., 2021). Future research should focus on *in vivo* models to directly validate these proposed mechanisms and their impact on tissue repair.

In addition to their role as miRNA sponges, circRNAs and lncRNAs are proposed to act as scaffolds for RBP complexes, coordinating the assembly and localization of RBPs to regulate inflammation and osteogenesis. Although these functions have been suggested by *in vitro* studies (Farooq et al., 2024; Huai et al., 2024), direct *in vivo* experimental evidence is lacking. Future research should prioritize the development of *in vivo* models to validate these proposed roles and elucidate the specific molecular interactions involved. For example, lncRNA/RBP complexes that control transcriptional and post-transcriptional processes regulate the activity of the nuclear factor kappa B (NF-κB) and transforming growth factor-beta (TGF-β) pathways, which manage inflammatory responses to injury (Li Y. J. et al., 2024; Lou et al., 2025; Wang B et al., 2025). Similarly, the Wnt/-catenin signaling pathway, which plays a vital role in osteoblast differentiation and bone formation, is regulated by circRNA and lncRNA-RBP assemblies that stabilize or activate osteogenic mRNA translation (He et al., 2020; Lv et al., 2024).

**FIGURE 3:**
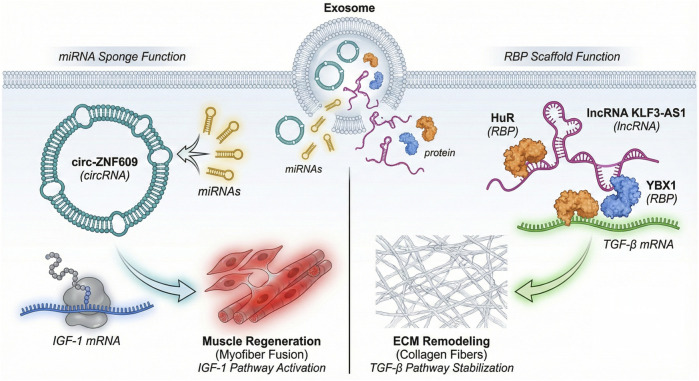
Exosomal circRNA/lncRNA mediates musculoskeletal repair via miRNA sponge and RBP scaffold functions. A schematic showing the dual functional mechanisms of exosomal circRNAs/lncRNAs (miRNA sponging and RBP scaffolding) in muscle regeneration and extracellular matrix remodeling.

Circ-ZNF609 has been proposed as an essential participant in repairing muscle and tendons as a representative molecule that engages with RBPs as miRNA sponges and participates in muscle and tendon repair by mediating myogenic differentiation and inflammatory signaling. Similarly, certain lncRNAs, such as KLF3-AS1, have been reported to be concentrated in exosomes and be involved in the suppression of pathological hypertrophy and the regenerative response in the muscle tissue (Chen et al., 2020; Wang L et al., 2025; Wen et al., 2022). These lncRNAs are stable, are highly encapsulated in exosomes, and can be transferred to damaged sites in the body, altering the gene expression networks that are pivotal to repair. CircRNAs and lncRNAs, therefore, serve as multifunctional molecular sponges and scaffolds, synthesizing signaling pathways to coordinate the musculoskeletal tissue regenerative milieu. Figure 3 is a visual representation of the canonical dual roles of exosomal circ-ZNF609 and lncRNA KLF3-AS1 in muscle regeneration and extracellular matrix remodeling (Harrell et al., 2019; Liu D et al., 2024).

### 3.2 Functions of RBPs in exosomes and their regulation of recipient cells

Exosomal RBPs are also used as key regulators of post-transcriptional genes in recipient cells during musculoskeletal injury repair. Hu antigen R (HuR) and Y-box binding protein 1 (YBX1) are key RBPs that are highly enriched in exosomes secreted by different forms of regenerative cells. They have been shown to mediate mRNA stability and translation, affecting protein synthesis that is required to repair tissues (Aborode et al., 2025; Youssef El Baradie and Hamrick, 2021). HuR interacts with the three primer transcripts in the three untranslated regions (AU-rich) of target mRNA and stabilizes transcripts that encode growth factors, cytokines, and extracellular matrix proteins involved in growth factor repair. YBX1 also acts similarly within the context of the mRNA fate; the molecule interacts with certain motifs to trigger or repress translation.

Exosome-associated RBPs can quickly stabilize injury signals and vary their cargo in response to recipient cell reparative needs. This high responsiveness is essential for regulating the rate of protein synthesis and the functional recovery of target cells that include myoblasts, osteoblasts, and fibroblasts (Fabbiano et al., 2020). Exosome proteomic studies demonstrate that RBPs dynamically change during injury and repair stages. These changes correlate with the nature of the regenerative response, suggesting that RBPs help fine-tune this process (Aborode et al., 2025; Sun et al., 2025). In addition, RBPs are implicated in the generation of ribonucleoprotein complexes that control alternative splicing, mRNA localization, and translation efficiency, with a coordination of complex gene expression programs required in inflammation resolution, cell proliferation, and matrix remodeling (Huang et al., 2025; Wozniak et al., 2020).

RNA interactome experiments with integrative proteomics approaches have identified large-scale regulatory networks of RBPs in exosomes, identifying nodes of interaction that assemble the interactions of several RNA molecules and signaling networks (Luo et al., 2024). These networks are dynamic in terms of time and space, and this is associated with the changing demands of the injured tissue microenvironment. Therefore, exosomal RBPs serve as cross-regional controllers that adjust the transcriptomes and proteomes of recipient cells, facilitating effective musculoskeletal healing and restoration of functions.

### 3.3 Multi-omics integration reveals systemic regulation of RNA–RBP networks

The repair of musculoskeletal trauma is regulated by RNA–RBP networks, which are increasingly understood at the mechanistic level through integrative multi-omics assays, including transcriptomics, proteomics, and exosome-specific omics. Proteomic profiling of exosomes pulled from injured musculoskeletal tissues facilitated by high-throughput RNA sequencing (RNA-seq) and proteomic profiling provides insight into the interaction between circRNAs, lncRNAs, miRNAs, RBPs, and their target mRNAs under exercise-induced stress conditions (Wang et al., 2019; Youssef El Baradie and Hamrick, 2021).

ExoCarta and other databases are helpful resources of exosomal RNA and protein cargo that can be used to identify regulatory molecules that play a role in musculoskeletal repair. The screening of candidate circRNAs, lncRNAs, and RBPs by mining these databases enables researchers to identify the participants in the injury and recovery phases that are differentially expressed or enriched in exosomes (Sun et al., 2025). A combination of these datasets with functional analyses of annotations and pathways identifies key signaling pathways such as IGF-1/PI3K/Akt, NF-2/TGF-2, and Wnt/3-catenin, in which RNA–RBP signaling interactions are perturbed (Engin, 2021; Yin et al., 2023).

Key network nodes have been functionally validated in animal models and gain- and loss-of-function experiments involving organoid systems that recapitulate musculoskeletal injury. They use silencing or overexpressing specific circRNAs or lncRNAs or altering the expression of RBP to verify their functions in the regulation of inflammation, cell growth, and extracellular matrix reorganization. These cell culture models show that tissue repair outcomes can be seriously influenced by the perturbation of RNA–RBP networks, highlighting their therapeutic potential (Zeng et al., 2020; Zhu et al., 2019).

Altogether, this multi-omics integration provides an overview of RNA–RBP regulatory signals during exosome-based musculoskeletal repair. The identified holistic view helps find new biomarkers and targets of therapies, which leads to more innovative precision medicine strategies that improve functional outcomes after musculoskeletal injuries. The major elements, repair outcomes, targets, and clinical promise of the exosome-dependent RNA–RBP network against musculoskeletal injury repair are reviewed in Table 2.

**TABLE 2:** Key components and functions of exosome-mediated RNA–RBP networks in musculoskeletal injury repair.

Core molecule type	Representative molecule	Target/Pathway	Repair effects	Clinical translation potential	Reference
Circular RNAs (circRNAs)	circ-ZNF609	Myogenesis-inhibitory miRNAs, NF-κB pathway	Promotes myogenic differentiation and inhibits excessive post-injury inflammation	Potential diagnostic biomarkers and therapeutic targets for injuries	Chen et al. (2020) ; Wang L et al. (2025)
Circular RNAs (circRNAs)	circ-Foxo3	Myogenesis-related miRNAs, PI3K/Akt pathway	Accelerates muscle/cartilage regeneration and reduces fibrosis	Candidate molecule for engineered exosome therapeutic vectors	Shi et al. (2022)
Long non-coding RNAs (lncRNAs)	KLF3-AS1	Pathological hypertrophy-related mRNAs, TGF-β pathway	Attenuates muscle pathological hypertrophy and promotes regenerative repair	Circulating exosomal diagnostic biomarker (similar to heart failure with reduced ejection fraction, Heart Failure with Reduced Ejection Fraction (HFrEF) application scenarios)	Wang L et al. (2025)
Long non-coding RNAs (lncRNAs)	lnc-TRPM2-AS	Th1/Th2 balance-related miRNAs	Regulates the local immune microenvironment at injury sites and promotes inflammation resolution	Intervention target for inflammation-associated injury repair	Hu et al. (2024)
RNA-binding proteins (RBPs)	HuR	Growth factor/cytokine mRNAs (3′UTR AU-rich elements)	Stabilizes repair-related mRNA and enhances protein synthesis	Functional regulator of exosome cargo	Aborode et al. (2025); Youssef El Baradie and Hamrick (2021)
RNA-binding proteins (RBPs)	YBX1	miR-223, myogenesis-related mRNAs	Mediates RNA sorting into exosomes and activates translational processes	Key target for exosome engineering modification	Aborode et al. (2025); Liu X. M. et al. (2021)
RNA–RBP complexes	circRNA–lncRNA–HuR complex	Wnt/β-catenin pathway	Promotes osteoblast differentiation and accelerates bone injury repair	Combined intervention target for bone regeneration therapy	He et al. (2020); Lv et al. (2024)
RNA–RBP complexes	lncRNA–RBP–YBX1 complex	Extracellular matrix remodeling-related genes	Regulates matrix synthesis during tendon/ligament repair	Potential intervention pathway for tendinopathy treatment	Liu X. M. et al. (2021); Sun et al. (2025)

Compiles the core components of RNA–RBP networks in musculoskeletal repair, including representative molecules, key pathways, and repair effects. The data are supported by preclinical and published studies, highlighting the value of clinical translation.

## 4 From biomarkers to engineered therapies: clinical translation of exosomal RNA–RBP networks in musculoskeletal repair

### 4.1 Exosomal RNA–RBP as biomarkers for musculoskeletal injuries

Exosomal RNA–RBP (RNA-binding protein) networks of biomarkers in musculoskeletal injuries are relatively promising in terms of early diagnosis and tracking of the occurrence of muscle strain, tendinopathy, and cartilage damage. Exosomes are extracellular vesicles released into the body fluids, such as plasma and synovial fluid, that contain a particular cargo of RNA and RBPs that display cellular states and pathological mechanisms. It has been shown that RBPs are selective in sorting of non-coding RNAs into exosomes, which affect intercellular interaction and tissue remodeling (Sun et al., 2023; Wang and Zhang, 2024). This preferential packaging suggests that injury-selective RNA–RBP profiles contained in exosomes could be effective, sensitive, and specific biomarkers. As an example, exosomal lncRNAs circulating in the bloodstream have been proposed as diagnostic signals in cardiovascular diseases, which demonstrates the validity of this strategy (Wang L et al., 2025). The stability of the exosomal RNA–RBP complexes in plasma and synovial fluid is beneficial in terms of using biomarkers because the vesicles prevent the destruction of their cargo by enzymes (Xu et al., 2019). New detection methods, such as high-throughput sequencing and mass spectrometry, have contributed to exosomal biomarker detection methods being highly sensitive and specific, allowing the differentiation between injury stages and severity (Romanowicz et al., 2024; Xing et al., 2023). In addition, exosomal RNA–RBP signatures could become tools used to predict the effectiveness of a post-hospital rehabilitation and identify overtraining syndromes by measuring current tissue stress and repair kinetics. However, the combination of exosomal biomarker profiles with longitudinal patient data would require clinical validation to help create strong associations with injury progression and recovery patterns. Composite biomarkers would be more diagnostic and prognostic when combined with molecular profiling and clinical parameters (Cheng et al., 2020; Rodrigues et al., 2023). To conclude, exosomal RNA–RBP networks are a novel frontier in musculoskeletal injury biomarker studies; they provide a noninvasive window into tissue remodeling and repair, which will revolutionize early diagnoses and individualized rehabilitation monitoring.

### 4.2 Design and application of engineered exosome therapies

Engineering exosomes to deliver pro-regenerative RNAs or anti-pathogenic RBPs holds promise for accelerating tissue remodeling and reducing fibrosis in musculoskeletal injuries. However, several challenges and limitations must be addressed to realize the full potential of this approach. For instance, achieving precise targeting of engineered exosomes to specific tissues remains a significant challenge. Current strategies, such as surface modification with targeting ligands, have shown promise in preclinical models but may face off-target effects and immunogenicity (Tian et al., 2023). Additionally, maintaining the stability of engineered exosomes during storage and *in vivo* delivery is crucial. Studies have demonstrated that modifications to enhance stability can sometimes compromise the therapeutic efficacy of the cargo (Liu et al., 2023). Furthermore, efficient delivery systems are needed to ensure that engineered exosomes reach the target tissues in sufficient quantities. Current delivery methods, including intravenous and local injections, have limitations in terms of bioavailability and distribution (Fan et al., 2024)

With engineered exosomes, therapeutic RNA species (e.g., circular RNAs, circ-Foxo3) that can modulate important signaling pathways in muscle and cartilage repair can be loaded (Shi et al., 2022; Taherpour et al., 2025; Wang et al., 2023). With the natural targeting and biocompatibility of exosomes, these molecular cargoes can be transported in a highly efficient fashion to injured tissues to improve endogenous repair pathways. The idea of molecular dressings or injectable delivery systems that include engineered exosomes presents a promising modality to generate localized and sustained delivery of therapeutic molecules to reduce inflammation and fibrotic scarring (Chen et al., 2022; Mérida-Cerro et al., 2025; Zhang F et al., 2023). The most recent developments in exosome modification methods, such as electroporation, ligand conjugation on surfaces, and genetic engineering of donor cells, have led to higher efficiency of loading, specificity of targeting, and stability of the therapeutic payload (Ma et al., 2025; Sadeghi et al., 2023). These technologies make it possible to design exosome-based therapeutics that are personalized based on the molecular pathology of a given musculoskeletal injury.

**FIGURE 4:**
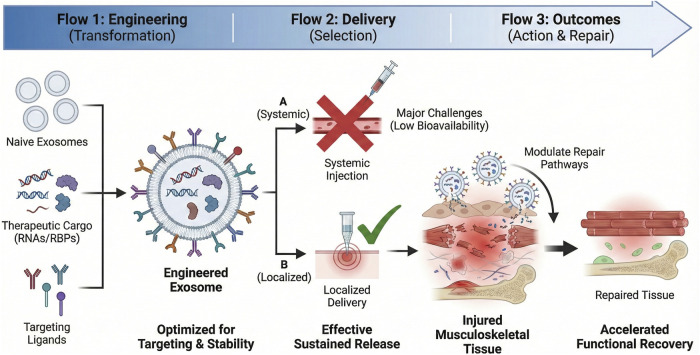
Engineering, delivery, and outcomes of exosome therapeutics in musculoskeletal injury. Schematic of the therapeutic workflow of engineered exosomes for musculoskeletal injury repair, covering the transformation of naive exosomes, the selection of localized versus systemic delivery systems, and the subsequent biological outcomes, including pathway modulation and tissue regeneration.

Key factors in the conceptual development of matchbox exosome therapies are the ability to produce exosomes on a large scale, to maintain their structural integrity when loaded, and to target them to muscle, tendon, or cartilage more effectively. Low safety profiles have been shown in immunogenicity and toxicity, and this could be translated into clinical practice (Sun et al., 2023; Yu et al., 2025). Moreover, combination approaches that incorporate enhanced exosomes alongside current rehabilitation guidelines can have a synergistic beneficial effect on functional recovery. Altogether, exosome therapeutics engineering and use are an innovative approach to regulating the RNA–RBP network in damaged musculoskeletal tissues, which promises to facilitate positive recovery and alleviate chronic disability (Li et al., 2025; Mancino et al., 2025). Figure 4 synthesizes the comprehensive workflow of exosome engineering, including the transformation of naive exosomes via cargo loading and surface modification, the selection of optimal delivery routes to overcome systemic bioavailability challenges, and the downstream outcomes of modulated repair pathways and functional recovery.

### 4.3 Translational pathways and challenges from laboratory to clinic

To translate exosomal RNA–RBP studies into clinical entities to repair musculoskeletal injuries, researchers must overcome several hurdles: standardization of production, control of dosage, immune compatibility, and regulatory compliance. To produce exosomes of high quality and with defined cargo profiles of RNA–RBPs, robust and scalable protocols that can enable reproducibility and purity are important (Kostyusheva et al., 2025; Sun et al., 2023). The exosome composition and their therapeutic efficacy can be altered by variability in donor cell sources, culture conditions, and isolation techniques. The identification of the correct dosage is rather complicated because the populations of exosomes are heterogeneous and the bioavailability of RNA–RBP cargo *in vivo* is dynamic. Immune reactions to exogenous exosomes, despite the overall low response levels, should be thoroughly considered, especially when exosomes with altered surface molecules or non-autologous systems are engineered (Grauwet et al., 2024; Li Y et al., 2024; Sadeghi et al., 2023).

Exosome-based therapies are currently in developmental regulatory review and must be supported by complete preclinical safety and efficacy data. The clinical studies would need to be multi-center clinical studies, based on the stratified sample groups of patients, and use a standardized outcome measure to confirm the therapeutic benefits and effectiveness of the treatment regimens. Future clinical trials should focus on larger cohorts and more diverse patient populations to better understand the therapeutic potential and limitations of engineered exosome therapies. Moreover, digital health integration alongside molecular diagnostics may support individualized exercise prescriptions along with molecular therapy, which will lead to a new chapter in musculoskeletal rehabilitation (Arora et al., 2024; Deering et al., 2024; Mamarabadi et al., 2025; Probyn et al., 2025).

**FIGURE 5:**
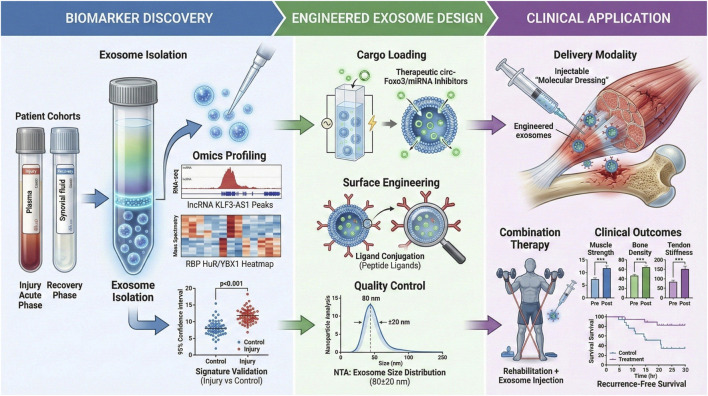
Translational workflow of exosomal RNA–RBP networks in musculoskeletal injury repair. Schematic of the translational workflow of exosomal RNA–RBP networks for musculoskeletal injury repair, covering diagnostic signature discovery, engineered exosome design, clinical translation pathways, and key challenges.

Future research options involve clarifying the mechanistic interactions between exercise-induced molecular signaling and exosomal RNA–RBP networks, devising predictive biomarkers of therapy responsiveness, and perfecting targeted delivery systems. Overcoming these translational challenges will be essential to achieve the clinical potential of exosome-based RNA–RBP modulation, through which musculoskeletal injury could be repaired and functionally restored. Figure 5 synthesizes the entire translational pipeline of exosomal RNA–RBP networks, including the discovery of diagnostic signatures by omics profiling, the design and optimization of engineered exosome therapeutics through the identification of pivotal crossroads, and obstacles to the clinical translation of these molecular networks into musculoskeletal injury repair.

## 5 Conclusions and perspectives

In summary, this review highlights the complex interplay between exercise-induced stress and musculoskeletal tissue adaptation, focusing on RNA expression changes and exosome-mediated communication. While the concept of exercise-induced RNA–RBP networks is promising, it is important to recognize that the majority of the current evidence is correlative or extrapolated from non-exercise contexts. Further experimental validation is needed to establish these networks as key players in adaptive remodeling and injury repair. The potential role of circRNAs, lncRNAs, and RBPs in mediating intercellular communication and tissue homeostasis is intriguing, but uncertainties remain. Future research should aim to validate these proposed mechanisms through targeted *in vivo* and *in vitro* experiments, which could provide more concrete insights into their roles in regeneration, inflammation, and osteogenesis. Considering the contradictory findings of the existing studies, it can be concluded that the exosome-mediated RNA–RBP axis is a biomarker reservoir and a treatment target at the same time. The use of unique exosomal RNAs and their corresponding RBPs is a positive step toward the early diagnosis of musculoskeletal trauma, which potentially enables clinicians to assess visible pathologic alterations related to the existence of numerous molecular transformations, prior to tissue damage manifesting itself. Moreover, exosome-based therapeutics represent a tremendous potential to enhance the outcome of rehabilitation due to the delivery of the particular molecular cargo that can control the signal pathways that become active during repair and regeneration. This diagnostic and curative power is a demonstration of the translational prospects of the application of the RNA–RBP network in clinical practice.

Despite these advances, transforming these molecular revelations into clinical practice is complex. The heterogeneity of musculoskeletal tissues and the variability across various modes of exercise and the complexity of RNA–RBP interactions necessitate a combination of multi-omics studies with rigorous functional validation. It shall be necessary to use transcriptomics with proteomics and epigenomics, plus *in vivo* and *in vitro* models, to identify the causation mechanism and maximize intervention plans. Moreover, the procedures for isolating and characterizing exosomes must be made uniform, reproducible, and comparable.

From a broader perspective, this integration of existing data contributes to the theoretical framework of exercise as medicine by providing the molecular basis upon which prescriptions can be tailored to specific situations. Not only does exosome-mediated RNA–RBP network elucidation represent a new direction in our knowledge of musculoskeletal biology, but it is also a move toward individualized exercise regimes with the best therapeutic results and the lowest injury rates. As more articles are published to elucidate these networks, the future of integrating molecular diagnostics into engineered therapeutics will instigate the rehabilitation paradigm to improve patient outcomes.

In general, the idea of exosome-driven RNA–RBP network development is a significant innovation in musculoskeletal research, bridging the gap between fundamental biology and clinical innovation. Future research should focus on addressing the key challenges in exosome engineering, such as improving targeting specificity, enhancing stability, and optimizing delivery systems. Additionally, comprehensive preclinical studies and well-designed clinical trials are needed to fully evaluate the therapeutic potential of engineered exosome therapies. These efforts will be crucial in translating the promising preclinical findings into effective clinical treatments for musculoskeletal injuries. This integrative paradigm will render it possible to have proper, molecularly precise interventions to gain a full understanding of and management of the changes that come with exercise. Ultimately, this development will help improve our ability to identify and treat musculoskeletal injuries in addition to cementing the role of exercise as a vital component of preventive and restorative medicine.
